# Immunolocalization of dually phosphorylated MAPKs in dividing root meristem cells of *Vicia faba*, *Pisum sativum, Lupinus luteus* and *Lycopersicon esculentum*

**DOI:** 10.1007/s00299-015-1752-6

**Published:** 2015-02-05

**Authors:** Konrad Krajewski, Aneta Żabka, Joanna Bernasińska, Karolina Matczak, Janusz Maszewski

**Affiliations:** 1 Department of Cytophysiology, Faculty of Biology and Environmental Protection, University of Lodz, ul. Pomorska 141/143, 90-236 Lodz, Poland; 2 Department of Thermobiology, Faculty of Biology and Environmental Protection, University of Lodz, ul. Pomorska 141/143, 90-236 Lodz, Poland; 3 Department of Molecular Biophysics, Faculty of Biology and Environmental Protection, University of Lodz, ul. Pomorska 141/143, 90-236 Lodz, Poland

**Keywords:** Mitogen-activated protein kinase (MAPK), Mitotic spindle, TEY motif, Phosphorylation cascade, Signaling

## Abstract

*****Key message***:**

**In plants, phosphorylated MAPKs display constitutive nuclear localization; however, not all studied plant species show co-localization of activated MAPKs to mitotic microtubules.**

**Abstract:**

The mitogen-activated protein kinase (MAPK) signaling pathway is involved not only in the cellular response to biotic and abiotic stress but also in the regulation of cell cycle and plant development. The role of MAPKs in the formation of a mitotic spindle has been widely studied and the MAPK signaling pathway was found to be indispensable for the unperturbed course of cell division. Here we show cellular localization of activated MAPKs (dually phosphorylated at their TXY motifs) in both interphase and mitotic root meristem cells of *Lupinus luteus*, *Pisum sativum*, *Vicia faba* (Fabaceae) and *Lycopersicon esculentum* (Solanaceae). Nuclear localization of activated MAPKs has been found in all species. Co-localization of these kinases to mitotic microtubules was most evident in *L. esculentum,* while only about 50 % of mitotic cells in the root meristems of *P. sativum* and *V. faba* displayed activated MAPKs localized to microtubules during mitosis. Unexpectedly, no evident immunofluorescence signals at spindle microtubules and phragmoplast were noted in *L. luteus*. Considering immunocytochemical analyses and studies on the impact of FR180204 (an inhibitor of animal ERK1/2) on mitotic cells, we hypothesize that MAPKs may not play prominent role in the regulation of microtubule dynamics in all plant species.

**Electronic supplementary material:**

The online version of this article (doi:10.1007/s00299-015-1752-6) contains supplementary material, which is available to authorized users.

## Introduction

The mitogen-activated protein kinase (MAPK) signaling pathway is comprised of three levels of sequentially activated modules, including mitogen-activated protein kinase kinase kinases (MAPKKKs), mitogen-activated protein kinase kinases (MAPKKs) and a group of effector serine/threonine kinases termed mitogen-activated protein kinases (MAPKs) at the bottom of a cascade (Taj et al. 2010). In mammals, 14 different MAPKs were found and classified into two separate subtypes: conventional and non-conventional. The former include ERK1/2 (extracellular signal-regulated kinase 1/2), p38, JNK/SAPK (stress-activated protein kinase) and ERK5 (Mishra et al. 2006; Cargnello and Roux 2011). Genome-wide analysis identified 16 members of MAPKs in a tomato. Transcripts of most analyzed genes were found in petal, stem, flower and fruit. However, expression of the majority of MAPKs was higher in reproductive organs than in vegetative ones (Kong et al. 2012). Up to 20 MAPKs have been identified in the genome of *Arabidopsis thaliana* so far (MAPK group 2002). Studies concerning MAPK signaling pathway are not limited to plants with sequenced genomes. Ortiz-Masia et al. (2008) investigated the function of MPK2 in *Pisum sativum* and found it expressed in vegetative and reproductive organs. Interestingly, roots exhibited the highest level of expression.

MAPKs are activated by dual phosphorylation of threonine and tyrosine in their conservative TXY motif (X is any amino acid). An activation loop of plant MAPKs may contain either a TEY (Thr-Glu-Tyr) sequence (kinases related to animal ERK) or a TDY (Thr-Asn-Tyr) sequence (distinctive of plants). Plant genomes studied so far have revealed no TPY (Thr-Pro-Tyr) or TGY (Thr-Gly-Tyr) sequences present in animal JNK and p38 kinase, respectively (Ulm 2003; Mishra et al. 2006). Interestingly, novel TQY (Thr-Gln-Tyr) phosphorylation motif of MAPKs described previously in nematodes was also found in legumes (Neupane et al. 2013a, b).

MAPK signaling pathway is a multifunctional cascade implicated in many cellular processes. MAPKs were found activated in response to biotic and abiotic stress, such as cold, salt, drought, wounding, chemical DNA damaging agents, UV and ionizing radiation (Ligterink et al. 1997; Tena et al. 2001; Ulm et al. 2001; Ulm 2003; Nakagami et al. 2005; Mishra et al. 2006; Pitzschke and Hirt 2006; Taj et al. 2010; Sinha et al. 2011). In all cases, activated MAPKs regulate gene expression via transcription factors. Thus, in animals, the main paradigm assumes inducible nuclear localization of MAPKs. On the other hand, in plants there is an ongoing controversy whether nuclear localization of these kinases is inducible or not. Depending on a plant model, some studies point to a constitutive nuclear localization of plant MAPKs, while others indicate an inducible recruitment to the nucleus (Šamajová et al. 2013).

Apart from environmental stress, MAPK signaling cascade is implicated in cell growth, cell cycle regulation, cell differentiation and development. Different types of MAPKs regulate cytoskeleton rearrangements (microtubules and actin filaments). Interestingly, MPK6, one of *A. thaliana* MAPKs, was also found to localize to the plasma membrane and secretory trans-Golgi network vesicles (Wrzaczek and Hirt 2001; Müller et al. 2010; Šamajová et al. 2013). Furthermore, Fernandez-Pascual et al. (2006) showed contribution of MAPKs to the symbiosis of *Bradyrhizobium* and *Lupinus albus*. The involvement of MAPK signaling pathway in other processes is still to be found.

At present, MAPKs are intensively studied for their interactions with microtubules. The mitotic spindle stability requires the activity of MAPKs in *Xenopus* egg extracts (Horne and Guadagno 2003). In *Arabidopsis*, MPK4 and MPK6 were found to localize to the phragmoplast during cytokinesis, ensuring an unperturbed cell division (Beck et al. 2011; Komis et al. 2011; Šamajová et al. 2013). Investigations in animals (Horne and Guadagno 2003) and plants (Krysan et al. 2002; Beck et al. 2011) indicate an essential role of MAPK signaling during mitosis. Proper microtubule dynamics require phosphorylation of MAP65 proteins (a group of microtubule associated proteins). This posttranslational modification diminishes the ability of MAP65 to bind microtubules and thus reduces microtubule bundling (Sasabe et al. 2006; Smertenko et al. 2006; Kosetsu et al. 2010). For example, MAP65 inactivation is indispensable during expansion of a phragmoplast and a defect in MPK4 results in microtubule bundling. Studies concerning MAPK signaling pathway mutants (*anp2, anp3* and *mpk4*) in plants show failures in both mitotic spindle formation and cytokinesis which lead to the formation of bi- and multinucleate cells (Beck et al. 2011).

Most studies on MAPK signaling pathway in plants were performed on model organisms, such as *A. thaliana*, *Nicotiana tabacum* and *Medicago sativa*. The role of plant MAPKs was investigated mainly in mutants and their localization (irrespective of their activity) was mostly determined by GFP-tagging or immunodetection of whole molecules. The abundance of MAPKs identified in *A. thaliana* and numerous processes they are involved in, raise the question of whether the range of cellular functions controlled by these kinases is invariable among plant species or if there are differences developed in the course of evolution.

Here, we show localization of activated MAPKs in plants representing two distinct families, i.e. Fabaceae (*Lupinus albus*, *P. sativum* and *Vicia faba)* and Solanaceae (*Lycopersicon esculentum*). To study this, commercially available antibodies against dually phosphorylated TEY sequence in animal p44/42 kinases were applied. However, due to potential cross-reactivity of these antibodies, it is possible to immunodetect both types of MAPKs, with TEY and TDY motifs. To the best of our knowledge, this is the first presentation of cellular localization of dually phosphorylated MAPKs in plants. Observations performed on selected species confirm the previously postulated role of activated MAPKs in the function of a mitotic spindle. However, a small number or lack of immunopositive mitotic cells in some species and differential response to FR180204, an inhibitor of animal ERK1/2 kinases (Ohori et al. 2007; Qian et al. 2008; Perrett et al. 2013), may suggest that not all plants engage MAPK signaling pathway to regulate microtubule dynamics during cell division.

## Materials and methods

### Material

Seeds of *Lycopersicon esculentum* var. *Faworyt, Vicia faba* subsp. *minor* var. *Nadwiślański, P. sativum* L*., Lupinus luteus* var. Mister and *Arabidopsis thaliana* were sown on wet filter paper in Petridishes and germinated for 3 days at 25 °C in darkness. For experiments, selected seedlings (with equally sized roots) were placed in Petridishes with water (control), with 80 µM FR180204 (Sigma) for 6 and 24 h or with 0.01 % (v/v) methyl methanesulfonate (MMS, Sigma) for 6 h.

### Western blotting

Proteins were extracted from root apical fragments (3 mm) with the use of P-PER Plant Protein Extraction Kit (Thermo Scientific) supplemented with Halt™ protease and phosphatase inhibitor cocktail (Thermo Scientific). The extracts were fractionated on NuPAGE^®^ Novex^®^ 4–12 % Bis–Tris gel (Invitrogen) and then blotted onto polyvinylidene fluoride (PVDF) membrane, 0.2-μm pore size (Invitrogen). A blocking buffer was prepared according to the vendor’s instructions (Chromogenic Western Blot Immunodetection Kit, Invitrogen). Dually phosphorylated TEY-type MAPKs were detected using monoclonal anti-phospho-p44/44 MAPK antibodies diluted 1:1,000 (Cell Signaling) and secondary goat anti-rabbit IgG antibody conjugated with alkaline phosphatase. The chromogenic reaction was run for 15 min. For total protein detection, PVDF membranes were stained with Ponceau S stain (Sigma) for 15 min.

### Co-localization of dually phosphorylated MPAKs and microtubules

Root apical fragments (3 mm) were fixed in 4 % (w/v) paraformaldehyde buffered with MTSB (50 mM PIPES, 5 mM EGTA, 5 mM MgSO_4_, pH 7.0) for 45 min, and then rinsed twice in PBS and transferred for 20 min (root fragments of *L. esculentum*) or for 45 min (the other plant species) to the citrate-buffered mixture (pH 5.0; 40 °C) containing 2.5 % (w/v) pectinase from *Aspergillus niger* (Sigma), 2.5 % (w/v) cellulase Onozuka R-10 from *Trichoderma viride* (Sigma), and 2.5 % (w/v) pectolyase Y-23 (MP Biomedicals). Next, root tips were rinsed twice in cold PBS (4 °C), squashed onto microscope slides (Super-Frost, Menzel-Gläser) in a drop of cold PBS, and placed on dry ice. After 10 min, cover slips were removed and the slides were washed with distilled water, followed by PBS and then air-dried. Macerated cells were permeabilized with 0.5 % (v/v) Triton X-100 for 15 min, preincubated in the blocking buffer (5 % w/v BSA, 0.3 % v/v Triton X-100, PBS) and then incubated overnight (4 °C) with primary monoclonal anti-β-tubulin antibodies (1:350, Sigma) dissolved in the antibody dilution buffer (1 % w/v BSA, 0.3 % v/v Triton X-100, PBS). Then the slides were washed in PBS and incubated at 25 °C for 90 min with secondary TRITC-conjugated anti-mouse IgG (whole molecule; 1:250, Sigma) dissolved in the antibody dilution buffer (1 % w/v BSA, 0.3 % v/v Triton X-100, PBS).

Next, the slides were incubated for 16 h (4 °C) with primary monoclonal anti-phospho-p44/44 MAPK antibodies (1:100, cell signaling) dissolved in the antibody dilution buffer (1 % w/v BSA, 0.3 % v/v Triton X-100, PBS). Then, the slides were washed in PBS and incubated at 25 °C for 90 min with secondary FITC-conjugated anti-rabbit IgG (whole molecule; 1:350, Sigma) dissolved in the antibody dilution buffer (1 % w/v BSA, 0.3 % v/v Triton X-100, PBS). Nuclear DNA was stained with 5 µM DAPI (Sigma) for 15 min, and then the slides were washed in PBS. The specimens were mounted in ProLong^®^ Gold Antifade Reagent (Invitrogen).

Cells were photographed using a wide field fluorescence microscope (Eclipse E600W, Nikon) equipped with a filter for FITC (EX 465–495, DM 505, BA 515–555), TRITC (EX 540/2, DM 565, BA 605/55) and DAPI (EX 340–380, DM 400, BA 435–485). Images were acquired with oil immersion ×100/NA 1.3. Impact of FR180204 on microtubule dynamics in *L. esculentum* was studied by means of Leica Laser Scanning Confocal Microscopy (LSCM) platform using laser line UV 405 nm and laser line supercontinuum visible 552 nm. Images were acquired with oil immersion ×100/NA 1.4.

### Mitotic indices and phase indices analyses

1.5 cm long root apical fragments were fixed in Carnoy’s mixture (ethanol/glacial acetic acid, 3/1, v/v) for 1 h. Following fixation, the roots were rinsed three times in 96 % ethanol, rehydrated (70–30 % ethanol, distilled water), hydrolysed in 4 M HCl (1.5 h for *V. faba* and 1 h for the other plant species) and stained with Schiff’s reagent (pararosaniline). After 1 h of staining, the roots were rinsed 3 times in 27 mM SO_2_-water and distilled water. Root tips (1.5–2 mm long) were excised and squashed in a drop of 45 % (v/v) acetic acid onto slides using the dry ice method. After removing cover slips, the slides were plunged into 70 % ethanol, air-dried and mounted in Canada balsam.

### Measurements and statistical analysis

Pearson’s correlation coefficient (R) was estimated by means of Fiji Is Just ImageJ Software, co-localization threshold mode (Schindelin et al. 2012). RGB (red–green–blue) pictures were split for red and green channels before analyses. ROI (region of interest), selected on images of β-tubulin immunodetection, refers to mitotic microtubules.

Mean immunolabeling indices (including the values calculated for mitoses and for successive phases of mitoses) were evaluated based on the analyses of 4 roots (ca. 150 analyzed cells per root) for each plant species. Total labeling index expresses the ratio of TEY-immunopositive mitotic cells to all mitotic cells. The labeling index for each phase of mitosis indicates the ratio of TEY-immunopositive cells at a particular stage of mitosis to all cells at this stage of mitosis. To evaluate mean values of mitotic index and phase index, 5 roots (for *V. faba* and *P. sativum*) and 10 roots (for *L. esculentum* and *L. luteus*) per each plant species (ca. 1,500–2,500 cells per root) were analyzed in Feulgen-stained specimens. Statistical analysis was performed using STATISTICA 10 PL Software. Differences between groups were assessed by Student *t* test. A *p* value ≤0.05 was considered statistically significant.

## Results

### *V. faba*, *P. sativum, L. luteus* and *L. esculentum* display differences in molecular masses of MAPKs

**Fig. 1:**
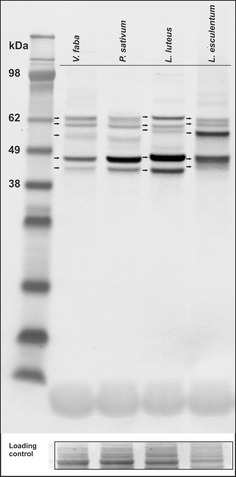
Immumoblotting analysis of dually phosphorylated MAPKs at Thr/Tyr in the whole-cell extracts. Loading control represents the level of proteins with molecular mass ranging from 80 to 98 kDa, detected with Ponceau S stain. *Arrows* point to bands of interest

Western blot (WB) analyses of crude extracts derived from the root apical fragments were performed with the use of monoclonal antibodies detecting human p-44/42 kinases dually phosphorylated in their conservative TEY motif. These antibodies have been already applied successfully in *A. thaliana* (Beck et al. 2011; González Besteiro et al. 2011). Similar to previous data, apart from plant homologs of ERK1/2 kinases, a number of proteins matching alternative types of MAPKs were immunodetected. Although all studied plants, *V. faba*, *P. sativum*, *L. luteus* and *L. esculentum*, showed five characteristic groups of proteins, some differences in the band position and in the quantity of phosphorylated molecules were found between species. *V. faba* and *P. sativum* displayed similar molecular masses of MAPKs while some differences in the extent of phosphorylation were revealed between these two species. However, among Fabaceae, a slightly different pattern of WB bands was found for *L. luteus*. Interestingly, unlike legumes, the molecular masses of immunodetected proteins extracted from *L. esculentum* were different (Fig. 1). The obtained results indicate that MAPK molecules display variable molecular masses in evolutionarily distinct plants, however, slight differences may also appear within the same family.

High level of MAPKs phosphorylation in the control plants prompted us to investigate whether cutting the root tip before homogenization induced kinase activation. Apical fragments of *V. faba* roots were excised on dry ice before homogenization both in the control plants and in those exposed to genotoxic stress (treated with 0.01 % v/v MMS for 6 h). The control plants displayed similar level of MAPKs phosphorylation irrespective to whether or not they had been excised on dry ice before homogenization. Furthermore, increase in the phosphorylation of MAPKs was found after MMS treatment in both cases. Notably, the extract from non-stressed *A. thaliana* seedlings (loaded as a control) showed only two bands corresponding to MPK3 and MPK6 (Fig. S1).

### Not all plants display co-localization of activated MAPKs to spindle microtubules in their root meristem cells

**Fig. 2:**
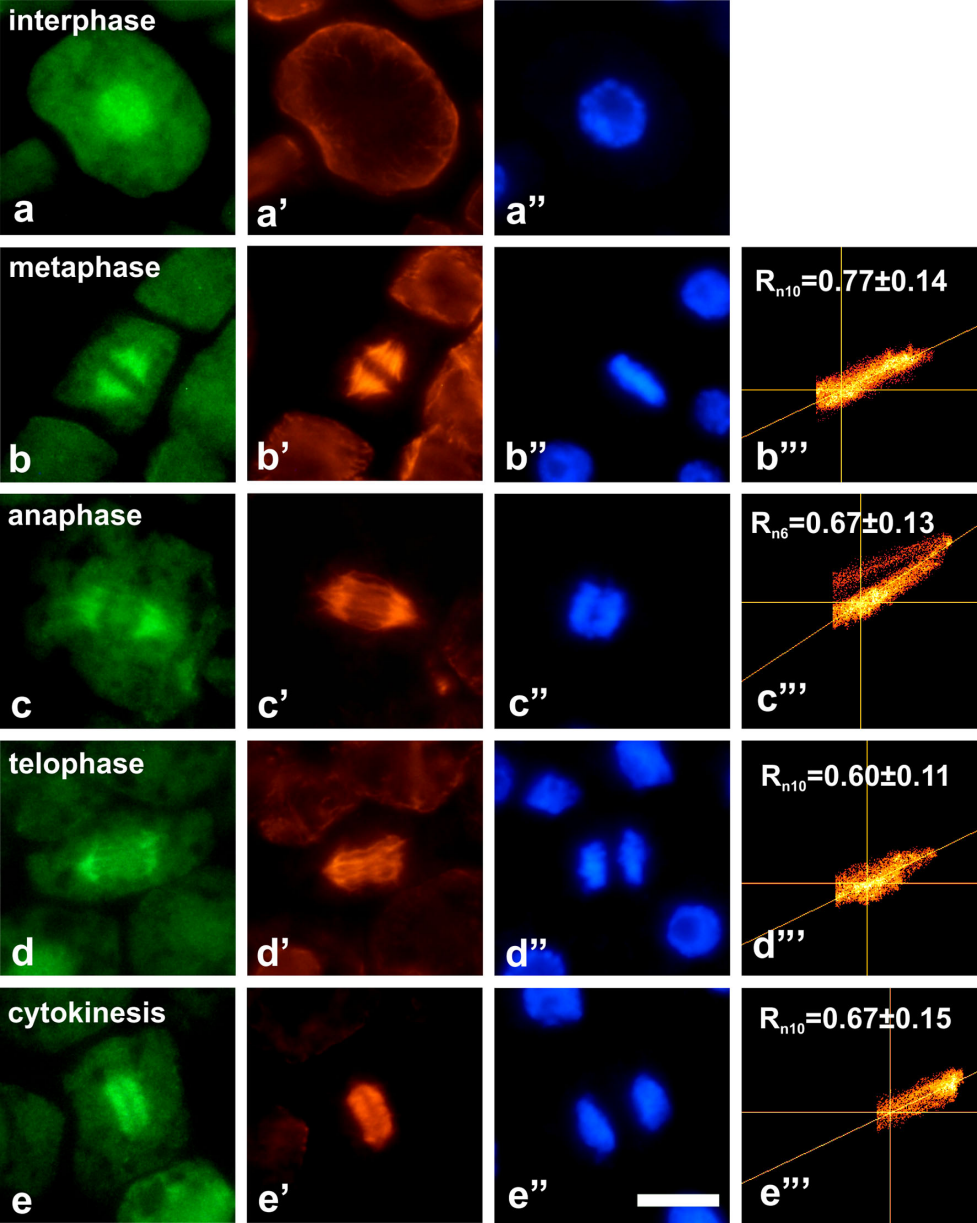
Co-localization of dually phosphorylated MAPKs at Thr/Tyr and β-tubulin in interphase cells and during successive stages of mitosis in *L. esculentum*. **a**–**d** Immunodetection of dually phosphorylated MAPKs, **a**’–**d**’ immunodetection of β-tubulin, **a**”–**d**” DAPI staining, **b**’’’–**d**’’’ scatter plots of representative images. *R* depicts mean values of Pearson’s correlation coefficient above a threshold, *n* indicates number of analyzed mitotic figures. *Bar* 10 µm

**Fig. 3:**
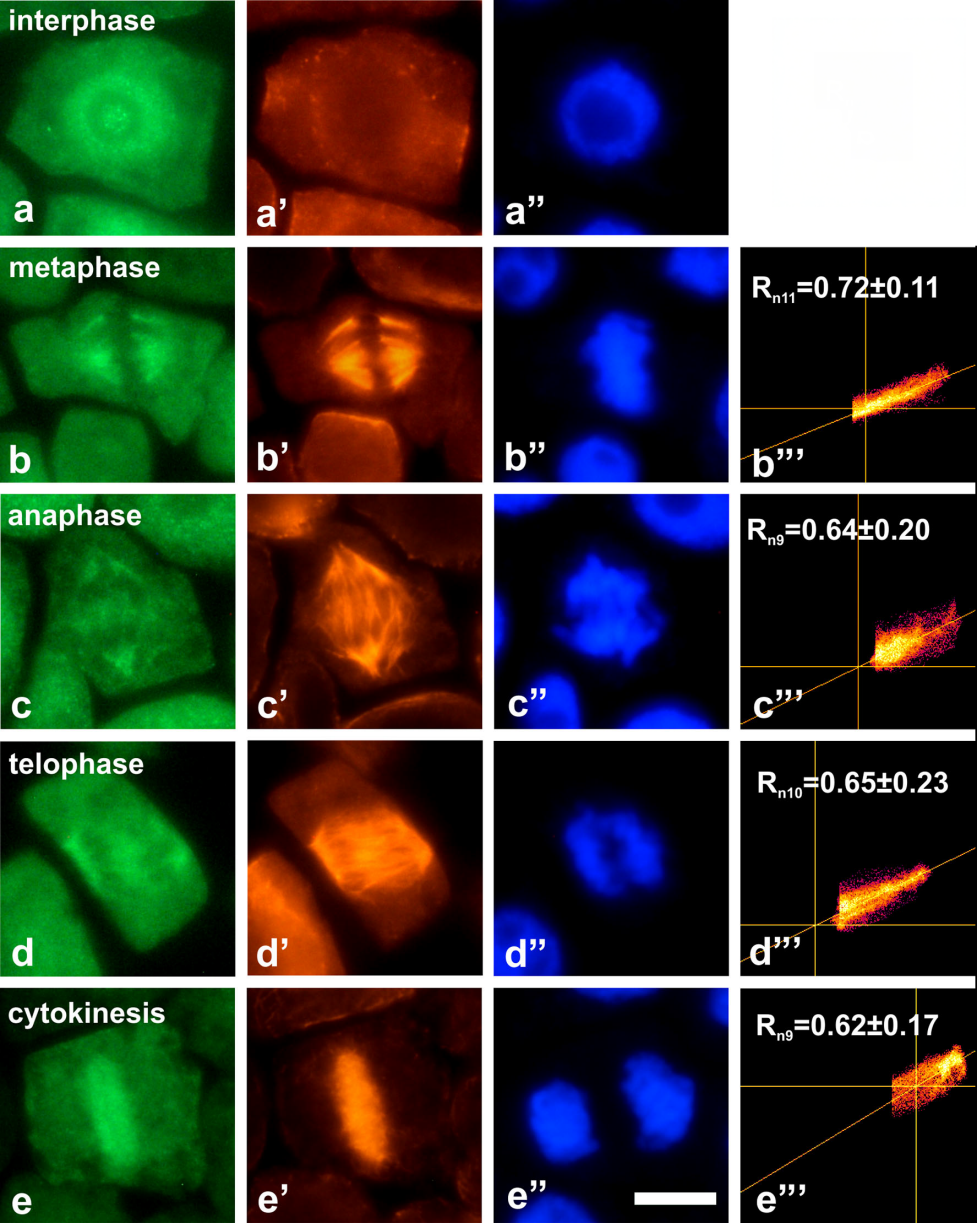
Co-localization of dually phosphorylated MAPKs at Thr/Tyr and β-tubulin in interphase cells and during successive stages of mitosis in *P. sativum*. **a**–**d** Immunodetection of dually phosphorylated MAPKs, (**a**’–**d**’ immunodetection of β-tubulin, **a**”–**d**” DAPI staining, **b**’’’–**d**’’’ scatter plots of representative images. *R* depicts mean values of Pearson’s correlation coefficient above a threshold, *n* indicates number of analyzed mitotic figures. *Bar* 10 µm

**Fig. 4:**
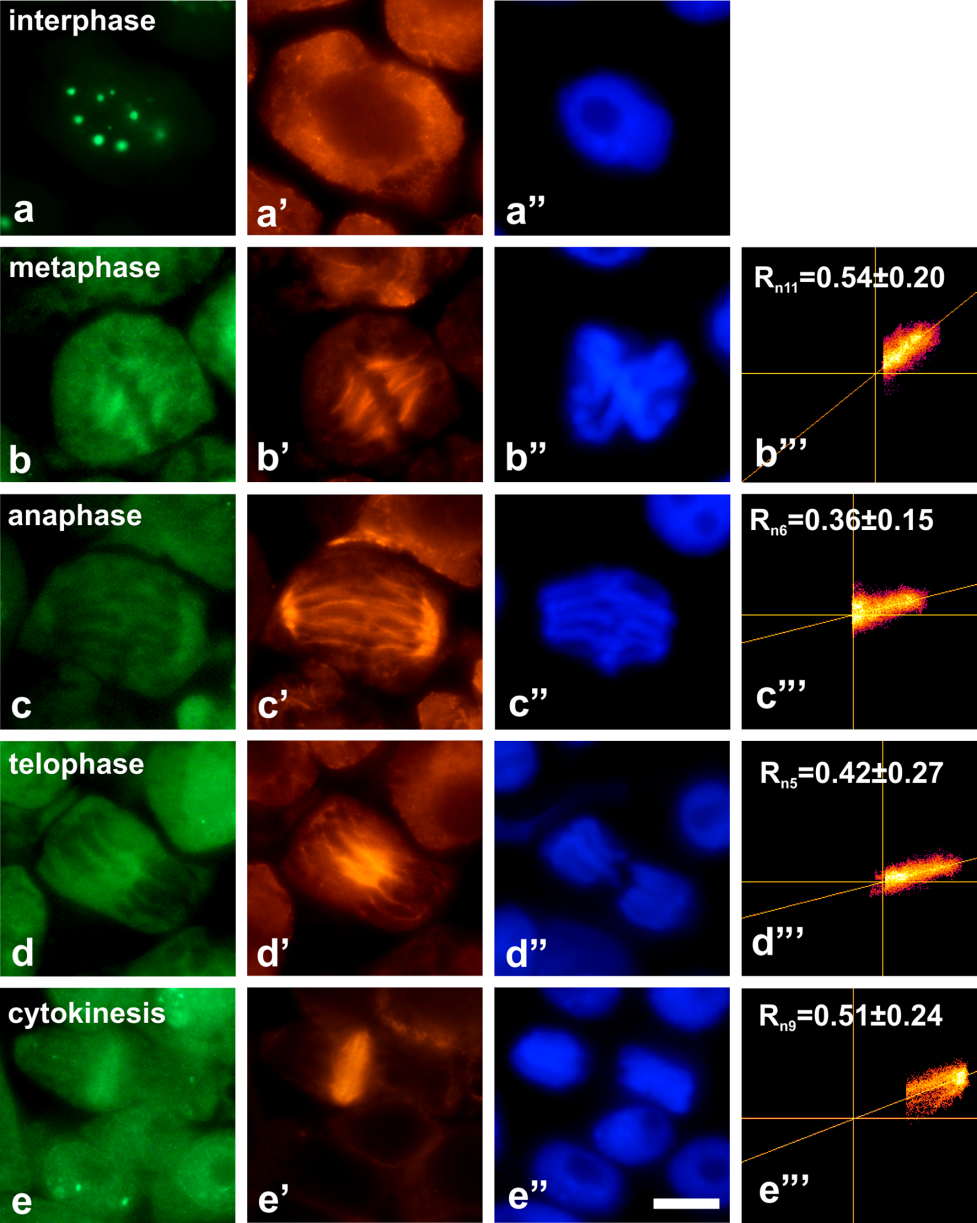
Co-localization of dually phosphorylated MAPKs at Thr/Tyr and β-tubulin in interphase cells and during successive stages of mitosis in *V. faba*. **a**–**d** Immunodetection of dually phosphorylated MAPKs, **a**’–**d**’ immunodetection of β-tubulin, **a**–**d** DAPI staining, **b**’’’–**d**’’’ scatter plots of representative images. *R* depicts mean values of Pearson’s correlation coefficient above a threshold, *n* indicates number of analyzed mitotic figures. *Bar* 10 µm

**Fig. 5:**
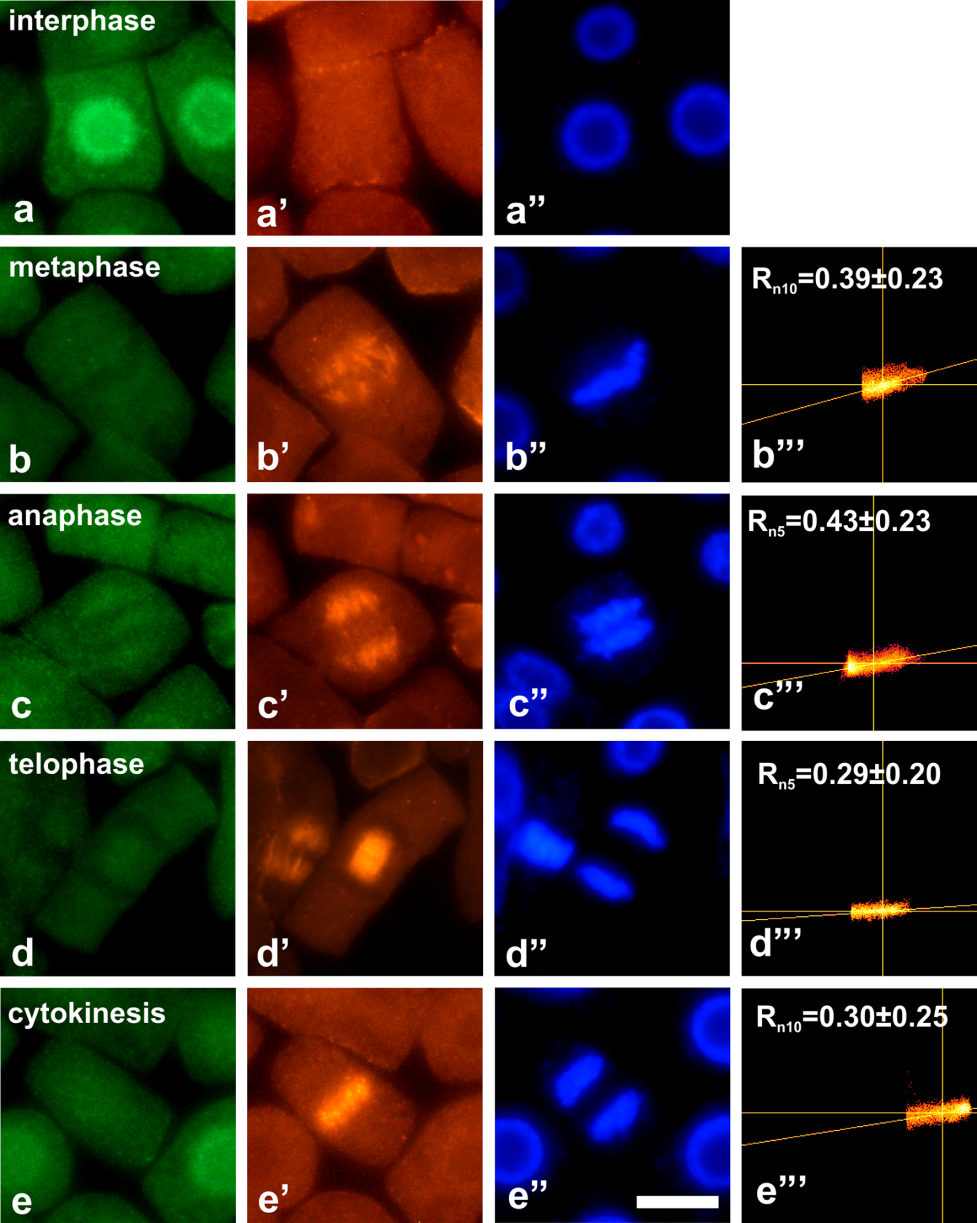
Co-localization of dually phosphorylated MAPKs at Thr/Tyr and β-tubulin in interphase cells and during successive stages of mitosis in *L. luteus*. **a**–**d** Immunodetection of dually phosphorylated MAPKs, **a**’–**d**’ immunodetection of β-tubulin, **a**”–**d**” DAPI staining, **b**’’’–**d**’’’ scatter plots of representative images. *R* depicts mean values of Pearson’s correlation coefficient above a threshold, *n* indicates number of analyzed mitotic figures. *Bar* 10 µm

Immunocytochemical analyses of root meristem cells clearly demonstrated nuclear localization of dually phosphorylated MAPKs in all studied species (Figs. 2, 3, 4, 5). In contrast to other plants, *V. faba* displayed immunofluorescence signals organized in regularly shaped foci (Fig. 4).

**Fig. 6:**
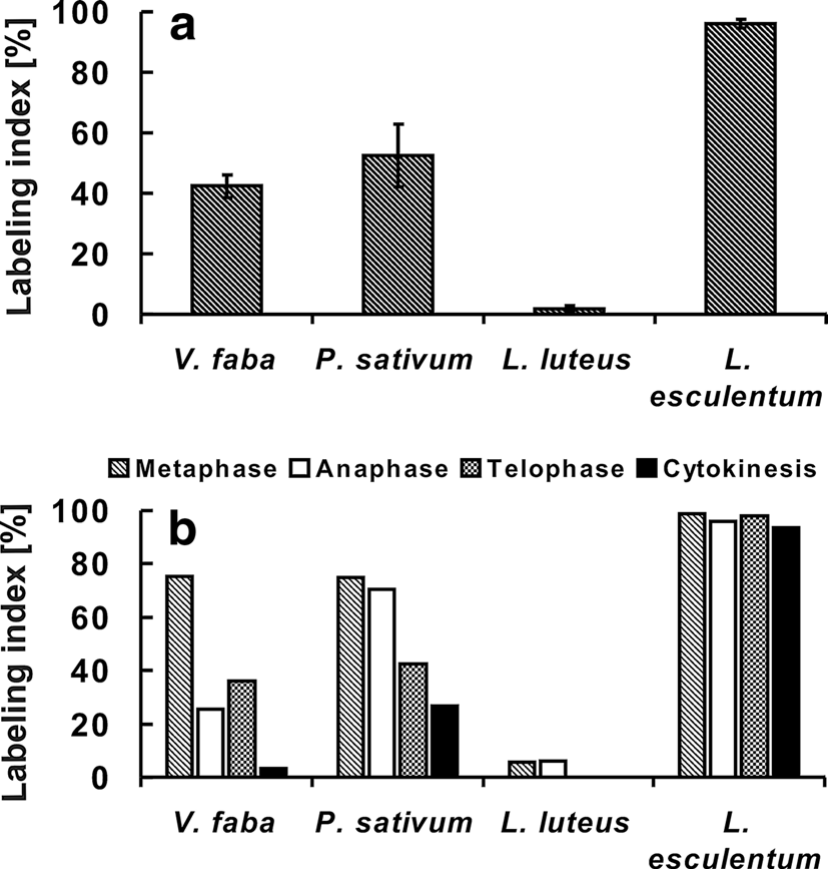
Labeling indices (%) estimated for root meristem cells stained with anti-phospho-p44/42 (Thr/Tyr) antibodies. **a** Total labeling indices, **b** labeling indices in the individual phases of mitosis

The most evident co-localization of dually phosphorylated MAPKs and mitotic microtubules was found in *L. esculentum* (Fig. 2). In this case, immunolabeled mitotic cells reached 95 % (Fig. 6a). In almost all metaphase cells (99 %), strong fluorescence signals appeared at kinetochore microtubules. From anaphase (labeling index = 96 %) to telophase (labeling index = 98 %) immunofluorescence signals at kinetochore microtubules diminished successively due to microtubule shortening. During cytokinesis (labeling index = 94 %), immunofluorescence signals were found at a phragmoplast (Fig. 6b). Similar localization of dually phosphorylated MAPKs was found in *P. sativum* (Fig. 3); however, the percentage of immunolabeled mitotic cells declined to 42 % (Fig. 6a). In this case, immunolabeled cells in metaphase reached 75 %, in anaphase 70 %, in telophase 42 % and during cytokinesis only 26 % (Fig. 6b). Similar value of the labeling index (53 %) was found for root meristem cells of *V. faba* (Fig. 6a); nevertheless, co-localization of activated MAPKs to spindle microtubules seemed to be less unequivocal than in the former species (Fig. 4). Although the labeling index estimated for metaphase cells reached 75 %, it declined during successive stages of mitosis to 26 % in anaphase, 36 % in telophase, and 3 % in cytokinesis (Fig. 6b). Despite the fact that *L. luteus* showed no evident fluorescence signals at spindle microtubules and a phragmoplast (Fig. 5), some faint immunofluorescence could still be seen in 5–6 % of metaphase and anaphase cells (Figs. 5, 6b). Notably, the frequency of phospho-TEY-positive mitotic cells was lower than 2 % (Fig. 6a). Since phosphorylation of MAPKs appeared in the nuclei of *L. luteus* root meristem cells, lack of evident co-localization of activated MAPKs and spindle microtubules does not seem to be an artifact.

Mean values of Pearson’s correlation coefficient (*R*) indicate different degree of correlation between green and red channels in various plant species. The highest *R* values were obtained for *L. esculentum* (Fig. 2b’’’–d’’’) and *P. sativum* (Fig. 3b’’’–d’’’). Weaker correlation was found for *V. faba* (Fig. 4b’’’–d’’’) whereas the smallest *R* values were obtained for *L. luteus* (Fig. 5b’’’–d’’’).

Single-labeled control (detection of dually phosphorylated ERK1/2) and secondary antibodies control (simultaneous labeling with anti-mouse and anti-rabbit secondary antibodies applied after anti-β-tubulin primary antibodies) were used to assess bleed-through and exclude cross-reaction between secondary antibodies, respectively. Additional experiments indicate that dually phosphorylated MAPKs display localization similar to that of spindle microtubules (Fig. S2). Furthermore, secondary antibodies show no evident cross-reactivity, however, small non-specific background fluorescence for FITC channel was detected (Fig. S3).

### Responses to FR180204, an inhibitor of animal ERK1/2, differ among plant species

**Fig. 7:**
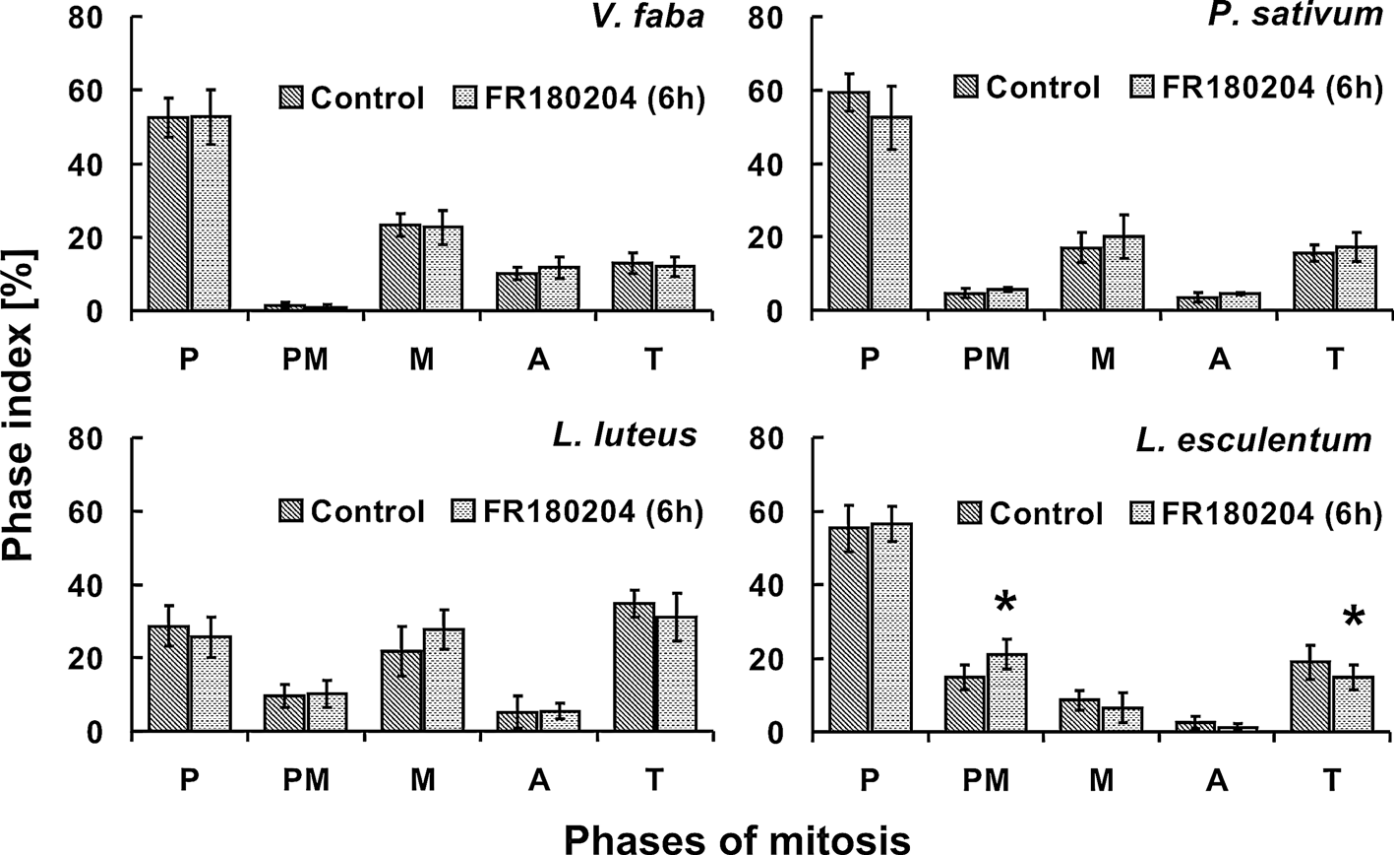
Percentage of cells in successive phases of mitosis (phase index) evaluated for plants incubated in water (control) or 80 µM FR180204 for 6 h. *Error bars* depict standard deviation. *Asterisks* indicate statistical significance (control versus FR180204, *p* value ≤0.05)

**Fig. 8:**
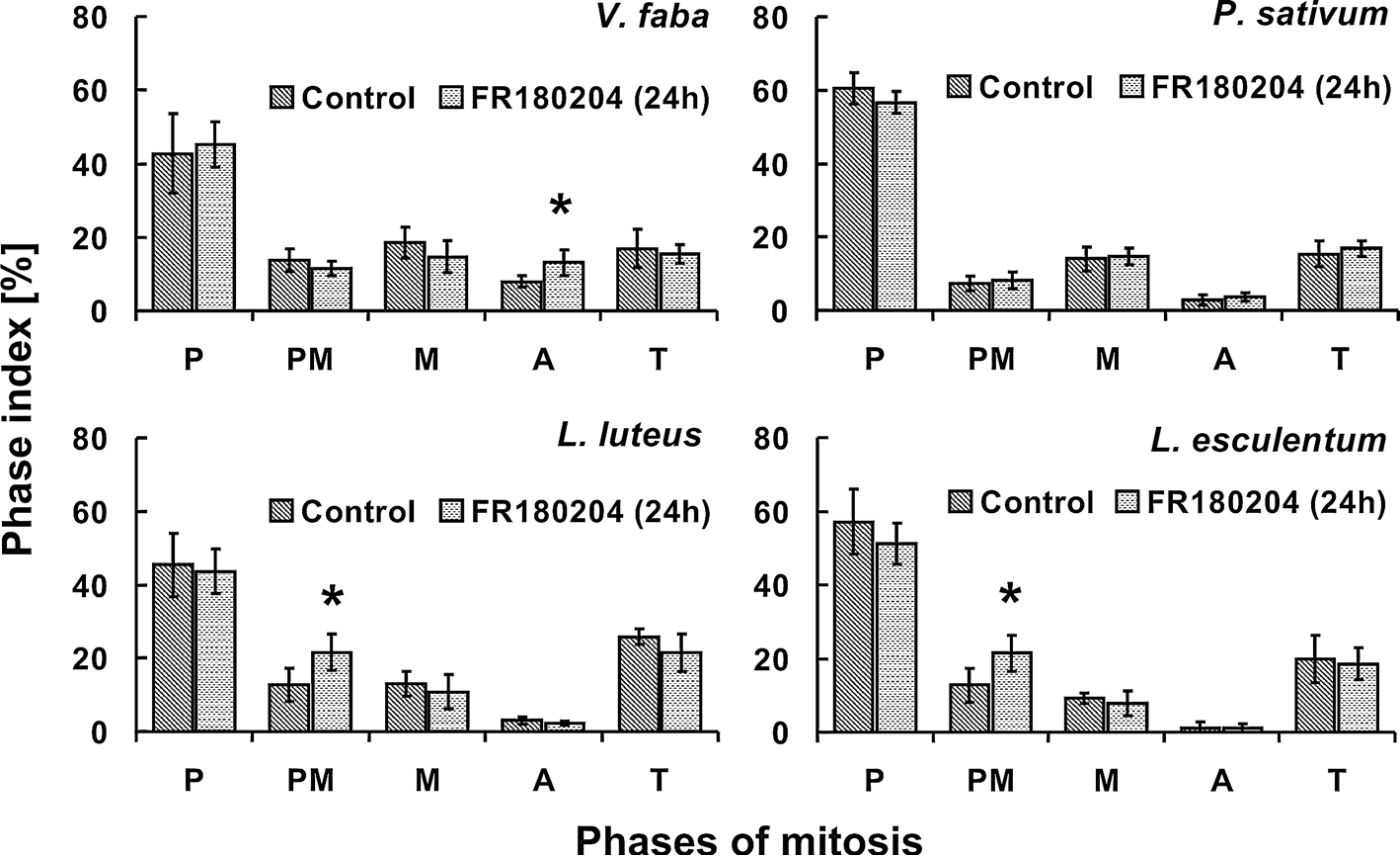
Percentage of cells in successive phases of mitosis (phase index) evaluated for plants incubated in water (control) or 80 µM FR180204 for 24 h. *Error bars* depict standard deviation. *Asterisks* indicate statistical significance (control versus FR180204, *p* value ≤0.05). *P* prophase, *PM* prometaphase, *M* metaphase, *A* anaphase, *T* telophase

The obtained data prompted us to investigate whether inhibition of MAPKs triggers differential effects in various plant species. To study this, plants were incubated in 80 μM FR180204, an inhibitor of animal ERK1/2, for either 6 or 24 h. Short, 6-h treatment affected mitosis only in the root meristem cells of *L. esculentum*. The presented data show a statistically significant increase in the number of prometaphases and a decrease in the number of telophases (Fig. 7). Seedlings incubated in FR180204 for 24 h displayed an increase in the number of prometaphases only (Fig. 8).

**Fig. 9:**
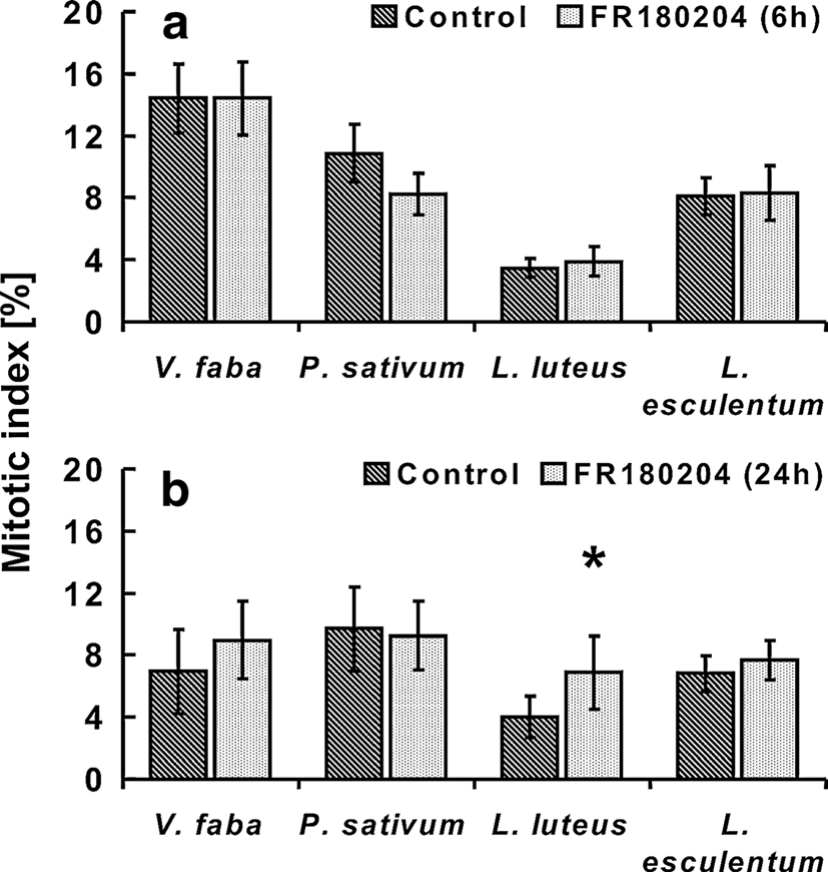
Percentage of mitotic cells (mitotic index) evaluated for different plant species. **a** Seedlings incubated in water (control) or 80 µM FR180204 for 6 h. **b** Seedlings incubated in water (control) or 80 µM FR180204 for 24 h. *Error bars* represent standard deviation. *Asterisks* indicate statistical significance (control versus FR180204, *p* value ≤0.05). *P* prophase, *PM* prometaphase, *M* metaphase, *A* anaphase, *T* telophase

Interestingly, despite the high percentage of immunolabeled metaphases and anaphases in the root meristem cells of *P. sativum* (Fig. 3b), the ERK1/2 inhibitor exerted no impact on the course of mitosis (Figs. 7, 8). The root meristems of *V. faba* and *L. luteus* displayed no changes in the percentage of cells at particular stages of mitosis upon 6-h incubation with FR180204 (Fig. 7); however, 24-h treatment brought about statistically significant increase in the percentage of anaphases or prometaphases, respectively (Fig. 8). Although, mitotic indices remained unchanged upon 6-h treatment with FR180204 (Fig. 9a), 24-h treatment brought about a statistically significant increase in the mitotic index in *L. luteus* (Fig. 9b).

**Fig. 10:**
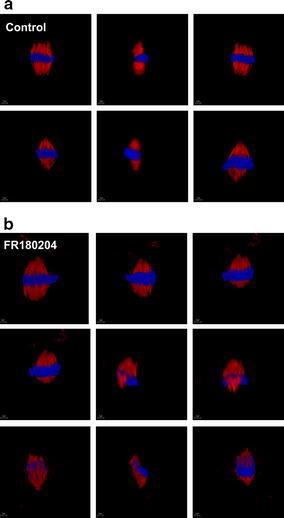
3-D images showing mitotic microtubules (*red channel*) in root meristem cells of *L. esculentum* during metaphase. **a** Control plants, **b** plants treated with 80 µM FR180204. *Blue channel* represents DAPI staining. *Across* different views of the same mitotic figure, *down* different mitotic figures (color figure online)

Since, *L. esculentum* displayed the strongest response to FR180204, impact of an ERK1/2 inhibitor on the mitotic spindle was studied by means of LSCM. Upon 6-h treatment, some dividing cells demonstrated problems with formation of the mitotic spindle. Microtubules seemed to display ectopic accumulation and improper direction of their polymerization (Fig. 10).

## Discussion

In this study, we applied commercially available antibodies against dually phosphorylated TEY motif in animal ERK1/2 kinases. WB analyses revealed five groups of proteins ranging from 40 to 62 kDa. Since kinases with TEY and TDY sequences displayed in *L. esculentum* different molecular masses, ranging from 42.72 to 45.51 kDa and from 51.98 to 70.55 kDa, respectively (Kong et al. 2012), we speculate that the applied antibodies, due to possible cross-reactivity, immunodetected in the studied plants two types of MAPKs, containing either TEY or TDY sequences in their activation loops. Based on the data presented by Kong et al. (2012), it is possible to make some predictions and attribute the protein bands immunodetected in *L. esculentum,* respectively, to MAPK1/MAPK2, MAPK10, MAPK14, MAPK15 and MAPK16. Furthermore, one of the immunodetected bands (≈43 kDa) in *P. sativum* could correspond to MPK2 (Ortiz-Masia et al. 2008). We demonstrated that MAPKs displayed different molecular masses in various plant species. The diversity in molecular masses was found not only between the plants representing distinct families but also within the same family, which seems to be an interesting aspect for further studies in the context of evolution and adaptation.

High level of MAPKs phosphorylation in the non-stressed plants, although not described previously, seems to be pertinent especially when we consider high expression of MAPKs in roots and carefully analyze the level of constitutive activity of MAPK proteins obtained during studies on environmental stress (Ortiz-Masia et al. 2008; Neupane et al. 2013a, b). Notably, our supplementary data indicated that cutting off apical root fragments before protein isolation did not induce detectable MAPKs-related stress. It is very probable that the low level of MAPKs phosphorylation in the control plants presented by others in environmental stress studies resulted from the short time of signal detection during Western blot analyses. Strong signal appearing rapidly in plants under stress conditions might have urged early termination of signal detection, thus preventing detection of phosphorylated MAPKs in the control plants.

Immunolocalization studies seem to be consistent with earlier data showing a prominent role of MAPKs during interphase and cell division (Bögre et al. 1999; Liu et al. 2004; Beck et al. 2010, 2011; Komis et al. 2011; Šamajová et al. 2013). By revealing stress-independent nuclear localization of activated MAPKs, our data strongly support the idea that these kinases control cell growth, differentiation and development not only influencing cytoskeleton dynamics but also regulating gene expression. However, the function of MAPK signaling pathways is still poorly understood in plants. Surprisingly, salt-induced relocation of nuclear pools of both SIMKK (stress-induced MAPKK) and SIMK (stress-induced MAPK) to cytoplasmic compartments was found recently (Ovečka et al. 2014).

The obtained data indicating evident co-localization of dually phosphorylated MAPKs and microtubules in metaphase, anaphase, telophase and during cytokinesis in *L. esculentum* and less pronounced in *P. sativum* and *V. faba* support previous studies pointing to essential role of MAPK signaling pathway in regulation of mitotic microtubule dynamics (Smékalová et al. 2014; Müller et al. 2010; Beck et al. 2010; Kosetsu et al. 2010).

Bögre et al. (1999) showed that a homolog of *V. faba* MMK3 kinase (one of *Medicago sativa* MAPK) was dispersed in metaphase cells, localized between chromosomes in early anaphase and confined to the phragmoplast during cytokinesis. This seems to contradict our results; however, the discrepancy may have appeared since we have immunodetected the whole spectrum of activated MAPKs whereas other studies were restricted to a particular type of kinase with its function possibly limited to a specific phase of mitosis (e.g. cytokinesis only). Moreover, it is probable that the immunofluorescence detected by Bögre et al. (1999) in metaphase cells of *V. faba* reflected both phosphorylated kinases localized to microtubules and inactive kinases dispersed in the cytoplasm.

Nevertheless, our results showed that the numbers of mitotic cells displaying co-localization of dually phosphorylated MAPKs to mitotic microtubules (if only detectable), differed depending on plant species. This, together with the lack of phospho-MAPKs immunofluorescence in mitotic microtubules of *L. luteus*, may indicate that in some plants the functioning of mitotic spindle is governed by other factors, e.g. CDK (cyclin-dependent kinase), which have already been found to cooperate with MAPKs in the regulation of microtubule dynamics (Smertenko et al. 2006). It is very probable that the mitotic microtubules in *L. luteus* are regulated mostly by CDKs.

Limited participation of MAPKs in the regulation of mitotic microtubules in some species may also be supported by differential response to FR180204, an inhibitor of animal ERK1/2. We assume that only a short, 6-h treatment reflects the direct impact of the inhibitor on microtubule dynamics. Thus, we hypothesize that in *V. faba* and *L. luteus* increases in the number of anaphases and prometaphases, respectively, upon prolonged 24-h treatment result from the effect of MAPKs inactivation on other proteins that control a cell cycle. Notably, it was previously found that inhibition of the MAPK signaling pathway in liver cancer cells triggered a decrease in CDK1 expression (Bessard et al. 2008). It was shown in plants that particular phases of mitosis were prolonged in cells with non-functional MAPK signaling pathway (Beck et al. 2011). Thus, similar numbers of prometaphases in *L. esculentum* obtained during 6- and 24-h treatments may suggest that the applied concentration of FR180204 extended the duration of prometaphases (due to microtubule disorders) rather than triggered a permanent block at this stage of mitosis. In turn, the increase in the number of telophases in seedlings of *L. esculentum* incubated with FR180204 for 24 h (compared to the plants treated for 6 h) may result from prolonged duration of telophases due to deregulation of cellular mechanisms controlling a cell cycle. Unlike severe mitotic microtubule disorders in MAPKs signaling mutants, moderate disturbances in the structure of mitotic apparatus upon FR180204 treatment were found in *L. esculentum*. It seems to support the transient effect of this inhibitor; however, at the same time it is probable that the applied concentration of FR180204 did not significantly affect plant MAPKs. Nevertheless, disturbances of the direction of microtubule polymerization during mitosis observed in our studies seem to be consistent with data presenting misaligned cell division planes in *yda* (one of MAPKKK) mutants (Smékalová et al. 2014).

Since the largest percentage of immunolabeled mitotic cells and clear response to 6-h treatment with FR180204 was found only in *L. esculentum*, it may implicate that in this species, contrary to the other studied plants, microtubule dynamics is mostly regulated by MAPKs. Although FR180204 seemed to have no impact on the function of mitotic spindle in *P. sativum* and *V. faba*, due to visible co-localization of MAPKs and mitotic microtubules, at least in about 70 % of metaphase cells, we hypothesize that these kinases may play a different role during cell division, other than controlling microtubule bundling. Furthermore, variable numbers of phospho-TXY-MAPKs immunopositive cells at successive stages of mitosis observed in *P. sativum* and *V. faba* seem to indicate that the time of MAPKs action during cell division might depend on the predominance of either anaphase A or B; both types are found in plants (Hayashi et al. 2007). Notably, contrary to the data presented by Müller et al. (2010), none of the plants examined in our study demonstrated an activated MAPK localization to preprophase band.

## Conclusions

The presented data indicate that the molecular masses of MAPKs differ not only in evolutionarily distinct plants but also between species belonging to the same family. Moreover, it appears that the functioning of a mitotic spindle may be independent of the activity of MAPKs in some plants. Thus, a hypothesis could be put forward that some plants phosphorylate MAP65, and thereby regulate the dynamic of mitotic microtubules independently of the MAPK signaling pathway (e.g. involving alternative kinases, such as CDKs). Participation of CDKs in hyperphosphorylation of MAP65 was previously shown (Smertenko et al. 2006). This idea might be also supported by the differential response to FR180204 among various plant species. A more detailed analysis needs to be performed to investigate the processes controlled by the MAPK signaling pathway and to shed new light on plant molecular evolution that may occur at the level of entire signaling pathways.

### **Author contribution statement**

KK conceived and designed research and analyzed data. KK, AŻ, JB and KM conducted experiments. KK and JM wrote the manuscript. All the authors read and approved the manuscript.

## Electronic supplementary material

Below is the link to the electronic supplementary material.

Supplementary material 1 (TIFF 13407 kb) Fig.S1 Immunoblotting analysis of dually phosphorylated MAPKs at Thr/Tyr in the whole-cell extracts from apical fragments of *V. faba* roots and seedlings of *A. thaliana*. Line 1 control plants of *V. faba*, line 2 MMS-treated plants of *V. faba*, line 3 control plants of *V. faba* (roots fragments excised on dry ice), line 4 MMS-treated plants of *V. faba* (roots fragments excised on dry ice), line 5 control plants of *A. thaliana*. Loading control represents the level of proteins with molecular mass ranging from 80 to 98 kDa, detected with Ponceau S stain

Supplementary material 2 (TIFF 15917 kb) Fig.S2 Immunodetection of dually phosphorylated MAPKs at Thr/Tyr in root meristem cells of *L. esculentum* (a), *P. sativum* (b), *V. faba* (c) and *L. luteus* (d). (a”-d”) secondary antibodies control (without primary antibodies). (a’-d’ and a’’’-d’’’) DAPI staining. Bar = 10 µm

Supplementary material 3 (TIFF 13245 kb) Fig.S3 Immunodetection of β-tubulin in root meristem cells of *L. esculentum* by means of mouse primary antibodies followed by simultaneous incubation in secondary anti-mouse and anti-rabbit antibodies conjugated with TRITC and FITC, respectively. (a) Fluorescence for FITC, (b) fluorescence for TRITC, (d) fluorescence for DAPI. Bar = 10 µm
