# Supplementary figures and images for: Immunolocalization of dually phosphorylated MAPKs in dividing root meristem cells of Vicia faba, Pisum sativum, Lupinus luteus and Lycopersicon esculentum
Source: Plant Cell Rep. 2015 Feb 5;34(6):905–17. doi: 10.1007/s00299-015-1752-6 (PMC4427623; doi:10.1007/s00299-015-1752-6)

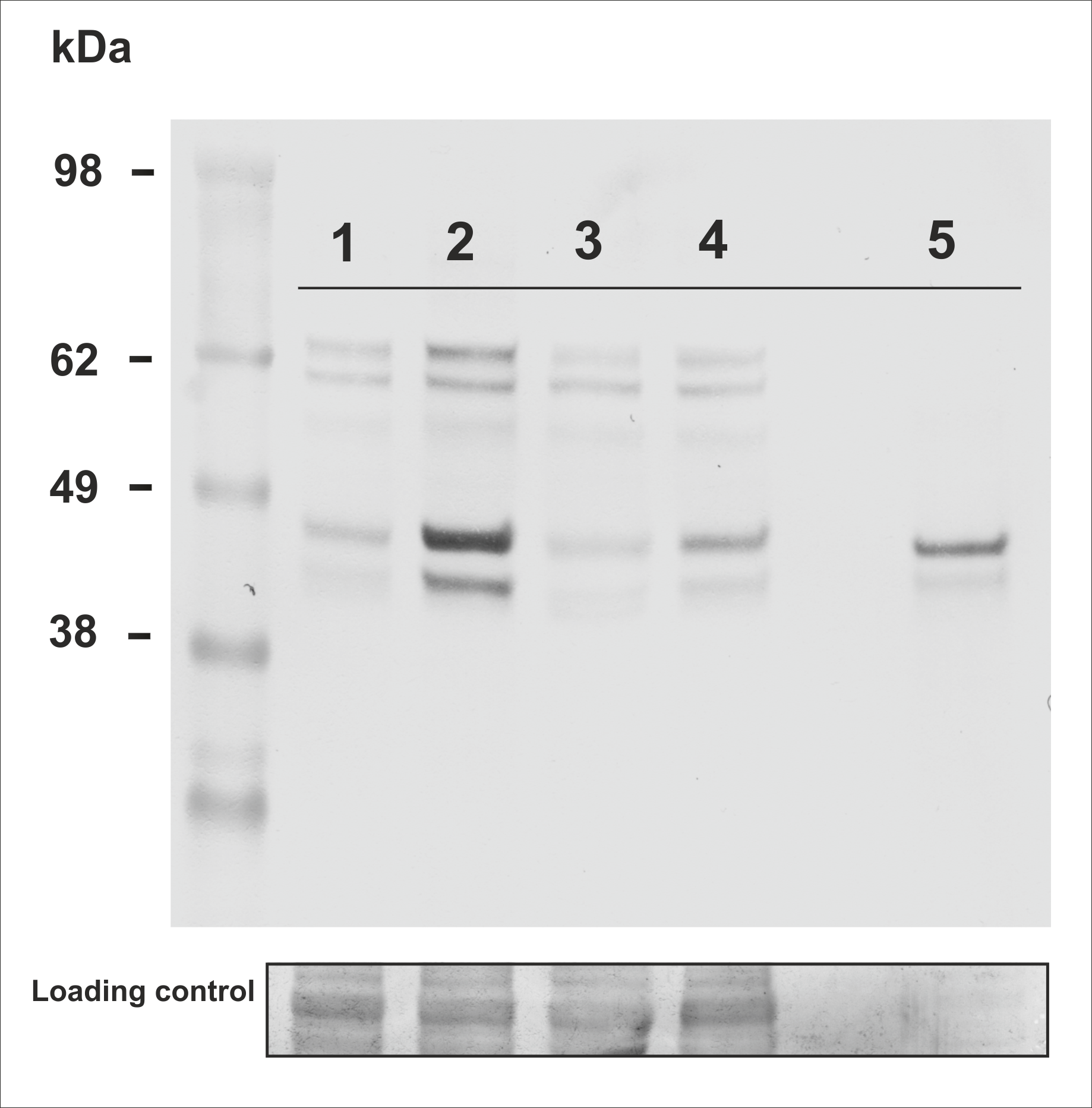

Supplement: Supplementary file 1 — Supplementary material 1 (TIFF 13407 kb) Fig.S1 Immunoblotting analysis of dually phosphorylated MAPKs at Thr/Tyr in the whole-cell extracts from apical fragments of V. faba roots and seedlings of A. thaliana. Line 1 control plants of V. faba, line 2 MMS-treated plants of V. faba, line 3 control plants of V. faba (roots fragments excised on dry ice), line 4 MMS-treated plants of V. faba (roots fragments excised on dry ice), line 5 control plants of A. thaliana. Loading control represents the level of proteins with molecular mass ranging from 80 to 98 kDa, detected with Ponceau S stain [file 299_2015_1752_MOESM1_ESM.tif]

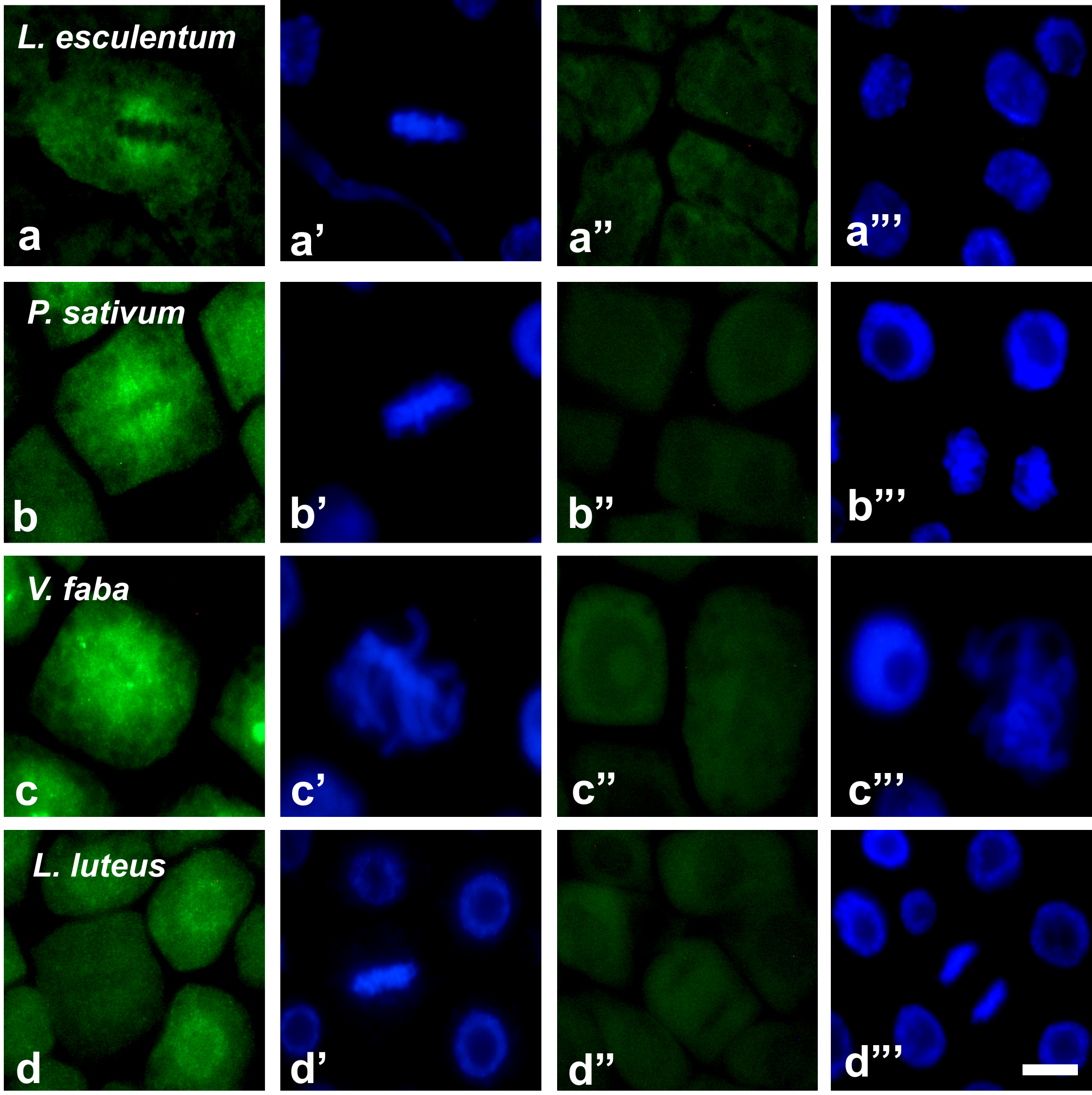

Supplement: Supplementary file 2 — Supplementary material 2 (TIFF 15917 kb) Fig.S2 Immunodetection of dually phosphorylated MAPKs at Thr/Tyr in root meristem cells of L. esculentum (a), P. sativum (b), V. faba (c) and L. luteus (d). (a”-d”) secondary antibodies control (without primary antibodies). (a’-d’ and a’’’-d’’’) DAPI staining. Bar = 10 µm [file 299_2015_1752_MOESM2_ESM.tif]

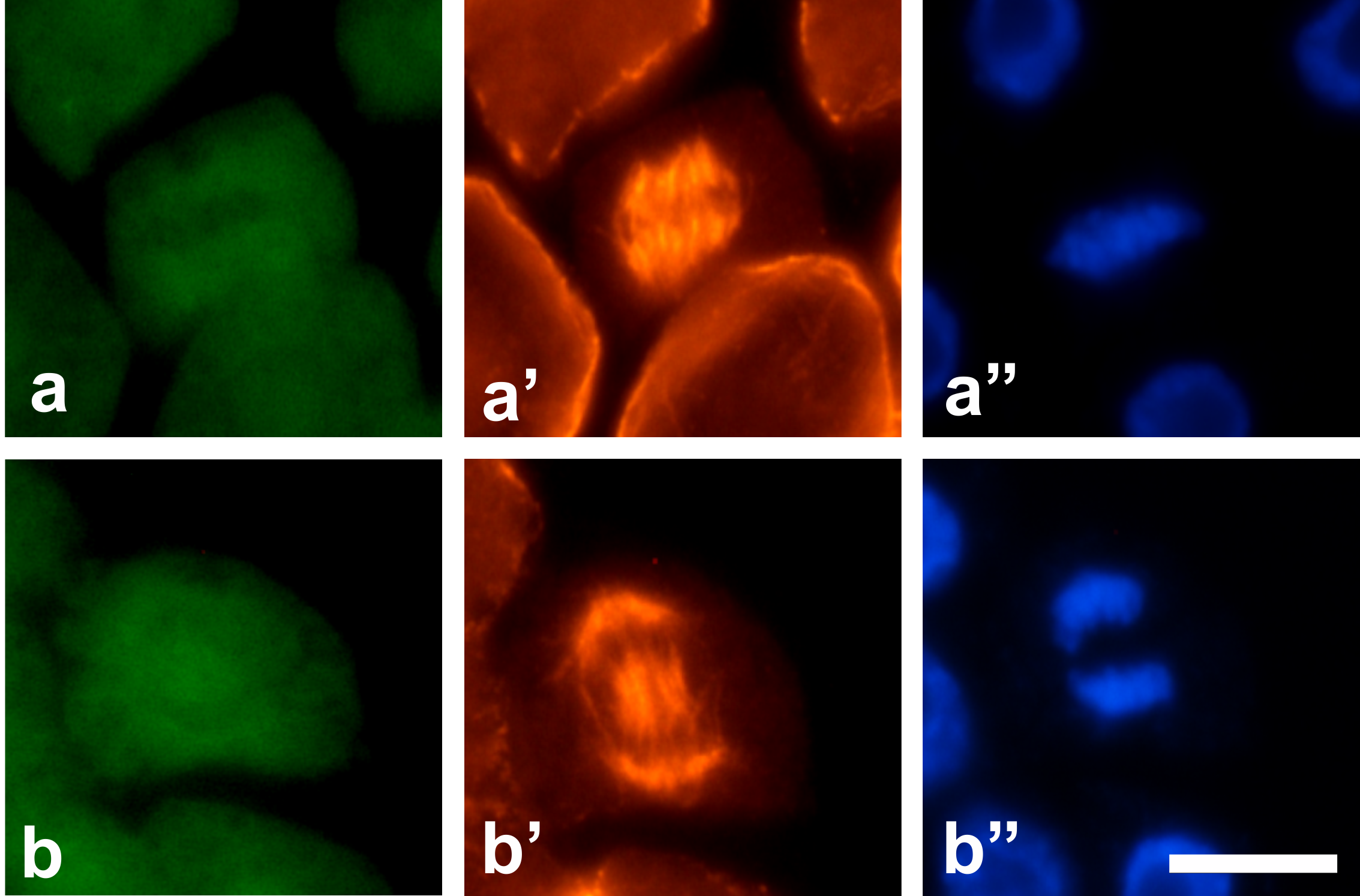

Supplement: Supplementary file 3 — Supplementary material 3 (TIFF 13245 kb) Fig.S3 Immunodetection of β-tubulin in root meristem cells of L. esculentum by means of mouse primary antibodies followed by simultaneous incubation in secondary anti-mouse and anti-rabbit antibodies conjugated with TRITC and FITC, respectively. (a) Fluorescence for FITC, (b) fluorescence for TRITC, (d) fluorescence for DAPI. Bar = 10 µm [file 299_2015_1752_MOESM3_ESM.tif]
